# Antibacterial and proteomic profiling of *Morus alba* extract against methicillin-resistant *Staphylococcus aureus*

**DOI:** 10.7717/peerj.20647

**Published:** 2026-01-23

**Authors:** Onrapak Reamtong, Thitiluck Swangsri, Tipparat Thiangtrongjit, Sompob Saralamba, Pakavadee Rakthong, Urusa Thaenkham, Naowarat Saralamba

**Affiliations:** 1 Department of Molecular Tropical Medicine and Genetics, Faculty of Tropical Medicine, Mahidol University, Bangkok, Thailand; 2 Mahidol Oxford Tropical Medicine Research Unit, Faculty of Tropical Medicine, Mahidol University, Bangkok, Thailand; 3 Faculty of Science and Technology, Rajabhat Suratthani University, Suratthani, Thailand; 4 Department of Helminthology, Faculty of Tropical Medicine, Mahidol University, Bangkok, Thailand

**Keywords:** *Morus alba*, MRSA, Proteomic analysis, Natural product, Antibacterial activity

## Abstract

**Background:**

Antimicrobial resistance, particularly from methicillin-resistant *Staphylococcus aureus* (MRSA), is a growing global health threat. Alternative therapies derived from medicinal plants are gaining attention for their potential to combat resistant pathogens. This study aimed to evaluate the antibacterial activity of *Morus alba* (white mulberry) extracts and investigate their action mechanisms using proteomic analysis.

**Methods:**

Leaf and stem samples of *M. alba* were extracted using both decoction and maceration techniques with water and ethanol as solvents. The antibacterial activity against MRSA was assessed through minimum inhibitory concentration (MIC), minimum bactericidal concentration (MBC), and time-kill assays. Chemical profiling of the most active extract was performed using liquid chromatography–quadrupole time-of-flight tandem mass spectrometry (LC-QTOF-MS/MS). Proteomic analysis was conducted to explore changes in bacterial protein expression after treatment.

**Results:**

The ethanol extract of *M. alba* stem exhibited the strongest antibacterial activity, with MIC values ranging from 0.3125 to 20 mg/mL and MBC values from 0.6250 to 40 mg/mL. A time-kill assay demonstrated that bacterial counts fell below the detection limit within 4 hours at four times the MIC concentration, based on three independent replicates. LC-QTOF-MS/MS profiling identified betulinic acid as the most abundant compound in the extract. Proteomic analysis revealed significant changes in MRSA protein expression, including upregulation of GlmU, N-acetylneuraminate lyase, and ribonuclease E, and downregulation of ribose-phosphate pyrophosphokinase and SecA. Pathway enrichment analysis suggested that the observed protein expression changes are consistent with enhanced N-acetylneuraminate catabolism and RNA polymerase activity, and suppression of protein export and fatty acid biosynthesis.

**Discussion:**

These findings highlight the strong anti-MRSA potential of *M. alba* stem extract and provide mechanistic insights into its antibacterial action. The extract disrupts critical metabolic and regulatory pathways in MRSA, supporting its potential development as a novel antimicrobial agent.

## Introduction

Antimicrobial resistance (AMR) is one of the most pressing public health threats of the 21st century, significantly compromising the prevention and treatment of bacterial infections. The accelerated emergence and spread of resistant pathogens hinder the effectiveness of current antibiotics and limit treatment options, contributing to increased morbidity, mortality, and healthcare costs (Friedman, Temkin & Carmeli, 2016). Among resistant bacteria, methicillin-resistant *Staphylococcus aureus* (MRSA) is of particular concern due to its resistance to multiple classes of antibiotics, posing substantial challenges to clinical management and infection control. Conventional antibiotics used to treat *S. aureus* infections include β-lactams such as oxacillin, cefazolin, and amoxicillin-clavulanate, as well as macrolides, tetracyclines, and fluoroquinolones. However, MRSA strains have acquired resistance to nearly all β-lactams through the mecA gene encoding penicillin-binding protein 2a (PBP2a), which reduces drug affinity (Vestergaard, Frees & Ingmer, 2019). Clinically, vancomycin, linezolid, daptomycin, and newer agents such as ceftaroline are used as last-line therapies against MRSA, yet reduced susceptibility and treatment failures have increasingly been reported (Dighriri et al., 2025). Given these concerns, there is an urgent need to discover and develop alternative antimicrobial agents. Natural products, particularly those derived from medicinal plants, offer promising candidates for novel antimicrobial therapies. Plant-derived antimicrobials are often considered safer than synthetic compounds (Abreu, McBain & Simões, 2012; Rajeh et al., 2010) and may act through mechanisms distinct from those of conventional antibiotics (Ahmad & Beg, 2001). Notably, nearly one-quarter of current pharmaceuticals are derived from plant-based compounds (Rates, 2001), underscoring the potential of medicinal plants in drug discovery. These natural compounds can provide alternative strategies to combat drug-resistant bacteria.

In traditional Thai medicine, numerous plants have been employed to treat infectious and dermatological conditions. For example, decoctions of *Lasia spinosa* (L.) Thwaites and pastes made from *Centella asiatica* (L.) Urb. are traditionally applied to treat skin ailments (Maneenoon et al., 2015). Ethnobotanical investigations and literature reviews have further highlighted the therapeutic potential of several plants, including *Ocimum gratissimum* L. var. *macrophyllum* Briq. (Viyoch et al., 2006), *Colocasia esculenta* (L.) Schott (Chakraborty et al., 2015), *Phyllanthus acidus* (L.) Skeels (Sowmya et al., 2018), *Annona squamosa* L. (Junsathian et al., 2022), *Pluchea indica* (L.) Less. (Chan et al., 2022), and *Morus alba* L. (Yiemwattana, Chaisomboon & Jamdee, 2018).

*Morus alba* has been widely used in traditional medicine systems and has demonstrated antimicrobial activity against various pathogens, including bacteria, fungi, and viruses. Different plant parts, leaves, stems, and fruits, are rich in bioactive compounds, particularly phenolic compounds, which are believed to contribute to its medicinal properties (Wang et al., 2012). Phytochemical screenings have revealed the presence of alkaloids, glycosides, flavonoids, saponins, tannins, and steroids in *M. alba* extracts (Faiz & Faiz, 2021). Among these, prenylated flavonoids such as kuwanon G, kuwanon H, and morusin have recently been reported to exhibit potent anti-MRSA activity at low micromolar or microgram-per-milliliter concentrations and to act synergistically with β-lactam or glycopeptide antibiotics (Škovranová et al., 2022; Zhu et al., 2024; Zuo et al., 2018). Previous phytochemical investigations have also shown that the chemical composition of *M. alba* extracts varies by plant part and solvent. Ethanol extracts, particularly those derived from stems, are typically rich in lipophilic and prenylated compounds such as oxyresveratrol, morusin, kuwanon G, and betulinic acid, whereas aqueous extracts contain more polar phenolics and glycosides (Batiha et al., 2023). Despite these findings, several important gaps remain. First, previous studies have mainly focused on identifying active compounds and evaluating their antimicrobial potency, while the molecular mechanisms underlying their antibacterial effects remain poorly understood. Second, the chemical composition of *M. alba* extracts varies by plant part and solvent, suggesting that different extracts may elicit distinct bacterial responses (De Oliveira et al., 2015; Chauhan et al., 2015). Understanding how these extracts affect bacterial physiology at the molecular level could inform the development of more effective antimicrobial therapies.

Proteomic approaches, particularly differential and functional proteomics, offer valuable tools for elucidating bacterial responses to antimicrobial compounds (Pandey & Mann, 2000). By analyzing changes in protein expression in bacteria exposed to plant extracts, proteomic studies can identify affected metabolic pathways, suggest action mechanisms, and uncover potential drug targets. Considering the pharmacological potential of *M. alba*, the variation in its bioactive compounds across plant parts and extraction methods, and the limited understanding of its molecular effects, this study was designed to addressed these gaps. Specifically, the objectives were to: (1) evaluate the antibacterial activity of aqueous and ethanolic extracts of *M. alba* leaves and stems against MRSA, and (2) investigate potential action mechanisms through comparative proteomic analysis.

## Materials and Methods

### Preparation of *M. alba* crude extracts

Leaves and stems of *M. alba* were collected on 8 November 2022 from a cultivated tree at a private residence in Bang Kruai, Nonthaburi, Thailand (approximately 13.82°N, 100.39°E), with permission granted by Dr. Naowarat Saralamba, the landowner, cultivator of the plant, and corresponding author of this study. A voucher specimen of *M. alba* has been deposited at the Mahidol University Herbarium, Thailand, under accession number PBM 006460.

All plant parts were thoroughly washed with distilled water, air-dried, and incubated at 60 ^∘^C for 72 hours. Dried plant materials were extracted using two solvents (distilled water and absolute ethanol) and two extraction methods (decoction and maceration). For decoction, 50 g of each plant part was soaked in 500 mL of distilled water, boiled at 100 ^∘^C for 10 min, filtered through Whatman No.1 filter paper, and lyophilized. For maceration, 50 g of plant material was soaked in 750 mL of absolute ethanol (100%) for 9 days at room temperature, filtered, and concentrated using a rotary evaporator.

To improve reproducibility and allow for comparison of extraction efficiency, each extraction was performed in two independent batches. Extraction yield was calculated as the percentage of dry extract relative to the starting plant weight. All crude extracts were reconstituted in dimethyl sulfoxide (DMSO) at a known concentration and stored at −20 ^∘^C until antibacterial assays. This standardized approach ensures consistent extract preparation across plant parts and solvents, providing a reliable basis for downstream bioactivity testing and proteomic analysis.

### Antibacterial activity assay

The antibacterial activity of *M. alba* extracts was evaluated against four bacterial strains: *Staphylococcus aureus* ATCC 25923, *S. aureus* MRSA252, *Escherichia coli* ATCC 25922, and *Pseudomonas aeruginosa* ATCC 27853. Bacteria were cultured overnight in Mueller Hinton Broth (MHB) at 37 ^∘^C in a shaking incubator and standardized to 10^8^ CFU/mL using a 0.5 McFarland standard.

Disc diffusion assay was performed to provide a qualitative, rapid screening of antibacterial activity and to visualize inhibition zones around the extract-impregnated discs, complementing the quantitative MIC/MBC data. Bacterial suspensions were swabbed onto Mueller Hinton Agar (MHA) plates. Whatman Antibiotic Assay Discs were loaded with 20 μL of plant extract. Discs containing DMSO and 10 µg gentamicin served as negative and positive controls, respectively. Plates were incubated at 37 ^∘^C for 18 hours, and inhibition zones were measured.

Minimum inhibitory concentration (MIC) and minimum bactericidal concentration (MBC) were determined using the microdilution method in 96-well plates. Extracts were two-fold serially diluted (starting from 50% v/v) in MHB to a final volume of 100 μL per well. Controls included bacteria with MHB, bacteria with 4% DMSO, bacteria with absolute ethanol, bacteria with distilled water (solvent control), 10 µg gentamicin (positive control), and media alone (negative control). After 18 hours of incubation at 37 ^∘^C, resazurin was added to assess viability. MIC was defined as the lowest concentration showing no visible growth. To determine MBC, 50 μL aliquots from non-growth wells were plated on MHA and incubated for 24 h. MBC was the lowest concentration resulting in no colony growth.

Time-kill studies were conducted using the ethanol stem extract at MIC, 2 × MIC, and 4 × MIC concentrations. MRSA cultures were adjusted to 6 × 10^5^ CFU/mL in a total volume of five mL. Tubes with extract, DMSO, absolute ethanol, distilled water, and media alone served as test and control groups. Cultures were incubated at 37 ^∘^C, and 100 μL samples were collected at 0, 1, 2, 4, 6, and 24 hours. Samples were serially diluted, plated on MHA, incubated for 24 hours, and viable colonies (CFU/mL) were counted.

### LC-QTOF-MS/MS for metabolite profiling

Metabolite profiling of the active extract was performed using LC-QTOF-MS/MS in negative ionization mode as described by Wu et al. (2021b). Analysis was carried out using a UHPLC system (UltiMate 3000 RSLCnano; Thermo Fisher Scientific) with an Acclaim^®^ RSLC120 C18 column (100 × 2.1  mm, 2.2 µm, 120 Å). The mobile phases were 0.1% formic acid in water (A) and acetonitrile (B). The gradient elution was programmed as follows: 2% B (0–1 min), linear increase to 90% B (1–12 min), hold at 90% B (12–14 min), and return to 2% B (14–18 min). The flow rate was 0.4 mL/min, and column temperature was maintained at 35 ^∘^C. Injection volume was one μL (10 ppm). Mass spectrometric analysis was conducted using a TripleTOF 6600+ system (AB SCIEX) with the following electrospray ionization (ESI) parameters: ion source gas 1 (50), gas 2 (60), curtain gas (30), temperature (150 ^∘^C), and ion spray voltage (−4500 V). The TOF-MS scan range was 100–800 amu, and product ion scan range was 50–800 Da. Tentative compound identification was performed by MS/MS spectral matching against the NIST 2017 and Natural Products HR-MS/MS libraries, comprising ∼13,800 and ∼1,000 compounds, respectively (Zhu et al., 2024).

### Proteomic analysis

Proteomic analysis was conducted to characterize MRSA protein expression changes upon exposure to *M. alba* ethanol extract. MRSA cultures were treated with the extract at the half-maximal inhibitory concentration (IC50), while untreated MRSA and MRSA treated with an equivalent volume of DMSO (solvent control) were included to identify extract-specific changes in protein expression. Bacterial pellets were lysed using lysis buffer and ultrasonication, followed by centrifugation at 12,000 × g for 15 min at 4 ^∘^C. Protein concentrations were determined *via* Bradford assay. Proteins were separated by 12% SDS-PAGE and visualized using Coomassie Brilliant Blue G250. Gel bands were excised for in-gel digestion. Proteins were de-stained in 50% acetonitrile/50 mM ammonium bicarbonate, reduced with 4 mM dithiothreitol (DTT) at 60 ^∘^C, and alkylated with 250 mM iodoacetamide for 30 min in the dark. After dehydration with 100% acetonitrile, gels were rehydrated with 10 ng/μL trypsin in 50 mM ammonium bicarbonate for digestion.

Peptides were analyzed using a MicroTOF Q II mass spectrometer coupled with nano-LC (UltiMate 3000; Thermo Fisher Scientific) and an Acclaim PepMap RSLC C18 column (75 µm × 15 cm, NanoViper). The MS scan range was m/z 500–3,500. Mascot software (v2.3, Matrix Science) was used for peptide identification against a custom MRSA transcriptome database, with the following parameters: one missed cleavage, variable modifications (carbamidomethylation and oxidation), trypsin specificity, peptide mass tolerance (0.8 Da), fragment mass tolerance (0.8 Da), and a significance threshold of *p* < 0.05. Analyses were conducted with three biological replicates, applying a fold-change cutoff of 2, and a corrected *p*-value threshold of 0.05. Protein–protein interaction and pathway enrichment analyses were conducted using the STRING database.

## Results

### Antibacterial activity of *M. alba* extract

Leaves and stems of *M. alba* were extracted using both maceration and decoction methods. Decoction with distilled water produced a higher extraction yield, particularly for leaf extracts (13.26%) compared to maceration (2.65%) (Table 1). All extracts were dissolved in DMSO prior to antibacterial assay.

**Table 1:** Extraction yields of *M. alba* crude extracts.

Plant part	Maceration (ethanol)	Decoction (distilled water)
Leaves	2.65%	13.26%
Stem	0.65%	4.04%

Disc diffusion assays revealed that macerated extracts exhibited stronger antibacterial activity than decocted ones. The stem extract from maceration showed the greatest activity, with inhibition zones ranging from 8 to 19.5 mm (Table 2). In contrast, decocted extracts displayed limited or no inhibition.

**Table 2:** Inhibition zone (mm) of *M. alba* extracts.

**Plant part**	**Extraction method**	**Concentration (mg/mL)**	***S. aureus*** **(ATCC 25923)**	**MRSA (BAA 1720)**	***E. coli*** **(ATCC 25922)**	***P. aeruginosa*** **(ATCC 27853)**
Leaves	Maceration	500	8	0	0	0
Leaves	Decoction	100	0	0	0	0
Stem	Maceration	500	18	19.5	10	8
Stem	Decoction	200	0	10	0	0
Gentamicin	—	10 µg	20.5	23.5	22	19
DMSO	—	—	0	0	0	0

MIC and MBC values were determined for the stem extract prepared *via* maceration. The MIC for *S. aureus* and MRSA was 0.3125 mg/mL, while higher MICs were recorded for *E. coli* (10 mg/mL) and *P. aeruginosa* (20 mg/mL). Corresponding MBCs were 0.625 mg/mL for *S. aureus* and MRSA, 20 mg/mL for *E. coli*, and 40 mg/mL for *P. aeruginosa* (Table 3). The antibacterial activity of the stem extract remained stable after three months of storage at −20 ^∘^C.

**Table 3:** Minimum inhibitory concentration (MIC) and minimum bactericidal concentration (MBC) of *M. alba* stem extract.

Bacterial strain	MIC (mg/mL)	MBC (mg/mL)
*S. aureus* (ATCC25923)	0.3125	0.6250
MRSA (BAA1720)	0.3125	0.6250
*E. coli* (ATCC25922)	10	20
*P. aeruginosa* (ATCC27853)	20	40

### Time-kill kinetics

**Figure 1:**
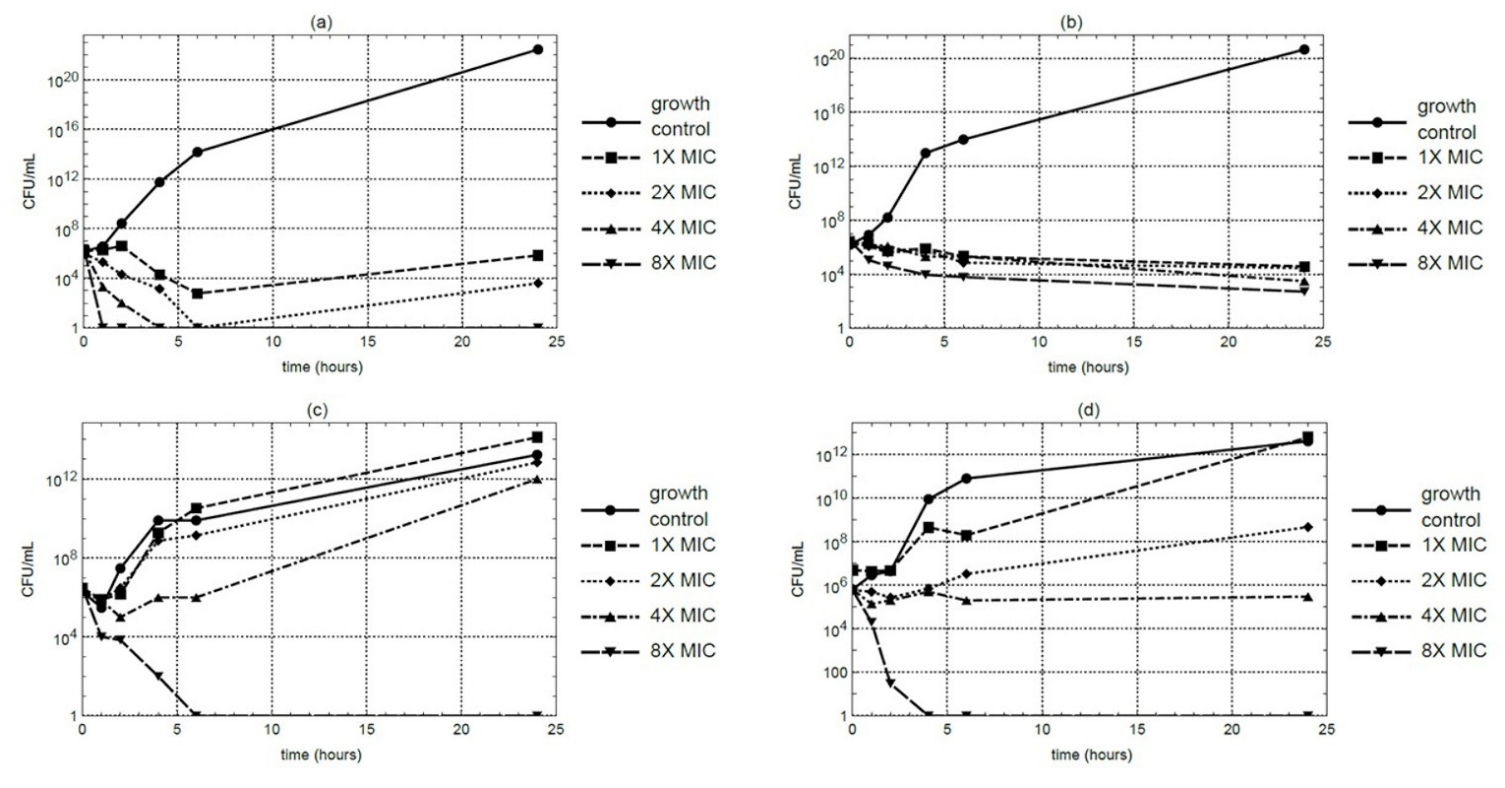
Time-kill curves of *M. alba* extract over a 24-hour period at 37 °C against (A) *S. aureus*, (B) MRSA, (C) *E. coli*, and (D) *P. aeruginosa*. Samples were collected at 0, 1, 2, 4, 6, and 24 hours for CFU quantification.

Time-kill kinetic studies were conducted using the macerated stem extract at MIC, 2 ×MIC, 4 ×MIC, and 8 ×MIC concentrations. At 4 ×MIC, *S. aureus* counts decreased to below the detectable limit within 4 hours under the assay condition. However, MRSA maintained residual viability even at 8 ×MIC. In contrast, *E. coli* and *P. aeruginosa* exhibited complete growth suppression within 6 and 4 hours, respectively, at 8 ×MIC (Figs. 1A–1D). Each time-kill assay was performed in triplicate, and results showed consistent trends across replicates.

### Metabolite profiling of *M. alba* stem extract

A total of 138 metabolites were identified *via* LC-QTOF-MS/MS after filtering for quality and abundance (File S1). Of these, 70 compounds have been previously reported to exhibit antimicrobial activity. The top ten bioactive metabolites, based on signal intensity and literature evidence, are listed in Table 4. Betulinic acid (BA) showed the highest abundance, followed by morin and oxyresveratrol. Several flavonoids were detected at substantial levels, including morin, the second most abundant compound in the extract, as well as luteolin and quercetin. Additionally, phenolic compounds such as esculin, esculetin, and p-coumaric acid were present, along with the flavonoid-related compound bavachin and the organic acid 2-isopropylmalic acid. These compounds were consistently detected across all replicates, supporting their prominence in the extract.

**Table 4:** Top 10 antimicrobial metabolites in *M. alba* stem extract.

No.	Found at mass	Area	Compound	References
1	455.3649	7,6860,000	Betulinic acid	Rodrigues et al. (2023), Hamion et al. (2024)
2	301.0447	6,4930,000	Morin	Nag, Dastidar & Chakrabarti (2021)
3	243.0741	4,4120,000	Oxyresveratrol	Joung et al. (2016)
4	285.0467	3,0720,000	Luteolin	Qian et al. (2020)
5	339.0785	2,1950,000	Esculin	Mokdad-Bzeouich et al. (2015)
6	301.0375	1,4080,000	Quercetin	Nguyen & Bhattacharya (2022)
7	177.0263	1,1250,000	Esculetin	Wu et al. (2021a)
8	323.1311	9949,000	Bavachin	Zhang et al. (2023)
9	175.0653	7742,000	2-Isopropylmalic acid	Ricciutelli et al. (2020)
10	163.0427	7577,000	*p*-Coumaric acid	Khatkar et al. (2017)

### Proteomic analysis of MRSA treated with *M. alba* extract

**Figure 2:**
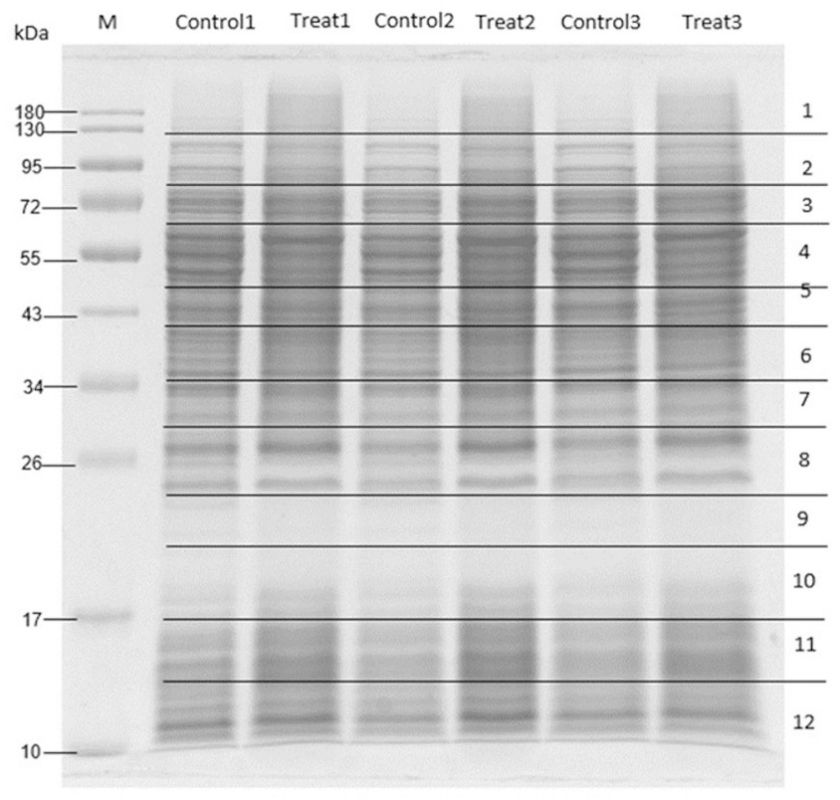
SDS-PAGE of MRSA proteins after treated with *M. alba* extract (IC50) and DMSO (0.1%). Proteins were resolved on a single gel and stained. The displayed image was converted to greyscale for clarity; the full-length, unprocessed blue-stained gel is provided in File S2.

To elucidate the antibacterial mechanism, MRSA was treated with *M. alba* stem extract at half the MIC and analyzed *via* proteomics. Untreated MRSA and MRSA treated with solvent controls were included as controls. SDS-PAGE followed by Coomassie staining confirmed protein presence (Fig. 2). LC-MS/MS revealed 41 differentially expressed proteins: 23 were upregulated (Table 5), and 18 were downregulated (Table 6). Among the upregulated proteins, bifunctional protein GlmU, N-acetylneuraminate lyase, and ribonuclease E showed the highest fold changes. In contrast, ribose-phosphate pyrophosphokinase, SecA translocase subunit, and ATP-dependent zinc metalloprotease FtsH were significantly downregulated.

**Table 5:** Upregulated proteins in MRSA after treatment with the *M. alba* extract.

**No.**	**Accession no.**	**Protein name**	**Score**	**M.W. (Da)**	**No. of peptides**	**% coverage**	**pI**	**Fold change**	**−log (*P*-value)**
1	A0A2T4RIN0	Bifunctional protein GlmU O	69	48,520	3	7.5	5.78	1.12	3.01
2	Q2G160	N-acetylneuraminate lyase	0	33,022	1	3.8	4.97	1.18	2.88
3	A0A3J8NWX5	Ribonuclease E	42	119,599	10	12.8	5.44	0.62	2.74
4	P0A0J0	RNA polymerase sigma factor SigA	42	49,020	7	15.3	4.95	1.12	2.65
5	A0A418J2F5	Coenzyme A biosynthesis bifunctional protein CoaBC	37	44,475	5	14.9	5.51	1.13	2.39
6	A0A2T4MDC9	Bifunctional protein GlmU	71	48,422	4	13.2	6.1	1.25	2.26
7	A0A505CYC4	Ribokinase	43	43,343	6	18.2	6.31	1.03	2.22
8	Q2FXM8	ATP-dependent 6-phosphofructokinase	98	34,818	9	25.8	5.63	1.3	2.14
9	A0A1Q8DEQ2	Coenzyme A biosynthesis bifunctional protein CoaBC	30	44,289	3	10	6.54	0.82	2.09
10	A0A2T4KPK4	CTP synthase	40	59,888	3	4.7	5.04	0.88	2.08
11	Q99QV3	Lactonase drp35	36	35,941	5	12.7	4.93	0.98	2.06
12	A0A418IM24	Riboflavin biosynthesis protein RibBA	31	43,601	5	13.4	4.83	1.12	2.01
13	P0A042	Penicillinase repressor	21	14,867	1	4.8	9.12	1.59	1.91
14	A0A133Q7U7	Aspartate-semialdehyde dehydrogenase	39	36,274	4	15.5	4.9	0.63	1.89
15	A0A830YRK5	Ribose-phosphate pyrophosphokinase	59	33,511	3	15.4	5.73	1.01	1.88
16	P0A017	Dihydrofolate reductase	135	18,239	6	37.7	5.87	0.63	1.81
17	A0A2T4QQF4	UDP-N-acetylmuramoyl-L-alanyl-D- glutamate–L-lysine ligase	94	54,148	8	22.9	5.43	0.6	1.73
18	O69174	Enolase	958	47,088	17	47.7	4.52	1.53	1.7
19	A0A2K4FF12	Aspartate-semialdehyde dehydrogenase	21	36,486	5	27.5	4.86	0.74	1.66
20	A0A418J779	CTP synthase	34	60,478	10	16.9	5.02	0.61	1.66
21	A6QIG7	Chemotaxis inhibitory protein	30	17,047	6	17.6	9.57	0.69	1.52
22	G5JJP7	Ribose-phosphate pyrophosphokinase	104	35,349	8	20.9	5.75	0.68	1.43
23	Q2FV99	Sortase A	28	23,526	5	25.7	8.85	0.62	1.38

**Table 6:** Downregulated proteins in MRSA after treatment with the *M. alba* extract.

**No.**	**Accession no.**	**Protein name**	**Score**	**M.W. (Da)**	**No. of peptides**	**% Coverage**	**pI**	**Fold change**	**−log (*P*-value)**
1	A0A7Z1N830	Ribose-phosphate pyrophosphokinase	82	32,777	4	22.4	5.73	–1.21	5.42
2	O06446	Protein translocase subunit SecA 1	234	95,900	16	22.3	5.11	–0.86	3.7
3	G5JHI9	ATP-dependent zinc metalloprotease FtsH	202	78,362	14	28.2	5.33	–0.80	3.62
4	A6QGC0	Serine/threonine-protein kinase PrkC	50	74,317	9	17.6	5.76	–0.93	3.09
5	A0A0H3JRU9	Pyruvate carboxylase	183	128,477	15	16.6	5.16	–1.33	2.89
6	Q2FZ08	Ribonuclease Y	48	58,477	6	16.6	5.18	–0.81	2.37
7	Q2FZQ3	Enoyl-[acyl-carrier-protein] reductase [NADPH] FabI	148	28,005	6	20.7	5.64	–1.81	2.31
8	A0A2T4LST6	Probable dual-specificity RNA methyltransferase RlmN	29	42,046	8	23.9	6.35	–0.62	1.97
9	A0A1Q8DD14	CTP synthase	93	59,862	12	20.9	4.93	–0.92	1.97
10	Q9F0R1	HTH-type transcriptional regulator SarR	63	13,660	2	22.6	9.34	–1.17	1.92
11	Q62386	Interleukin-17A	17	17,479	2	26.6	9.19	–1.38	1.91
12	A0A1X0U0N1	Probable dual-specificity RNA methyltransferase RlmN	39	41,477	6	17.1	6.77	–0.70	1.87
13	A0A0H3K9F2	Low molecular weight protein- tyrosine-phosphatase PtpA	60	17,479	3	26	4.63	–1.32	1.86
14	A0A2K4AHR4	Aspartate-semialdehyde dehydrogenase	30	36,344	4	16.7	4.85	–0.68	1.83
15	P0A0B7	Alkyl hydroperoxide reductase C	656	20,963	14	71.4	4.88	–0.82	1.7
16	A0A3A0VLJ1	CTP synthase	94	60,071	7	16.4	4.97	–0.78	1.67
17	I3LGZ3	Protein GPR15LG	18	9,208	4	10.4	10.62	–1.02	1.61
18	P59666	Neutrophil defensin 3	19	10,238	4	42.6	5.71	–0.70	1.45

**Figure 3:**
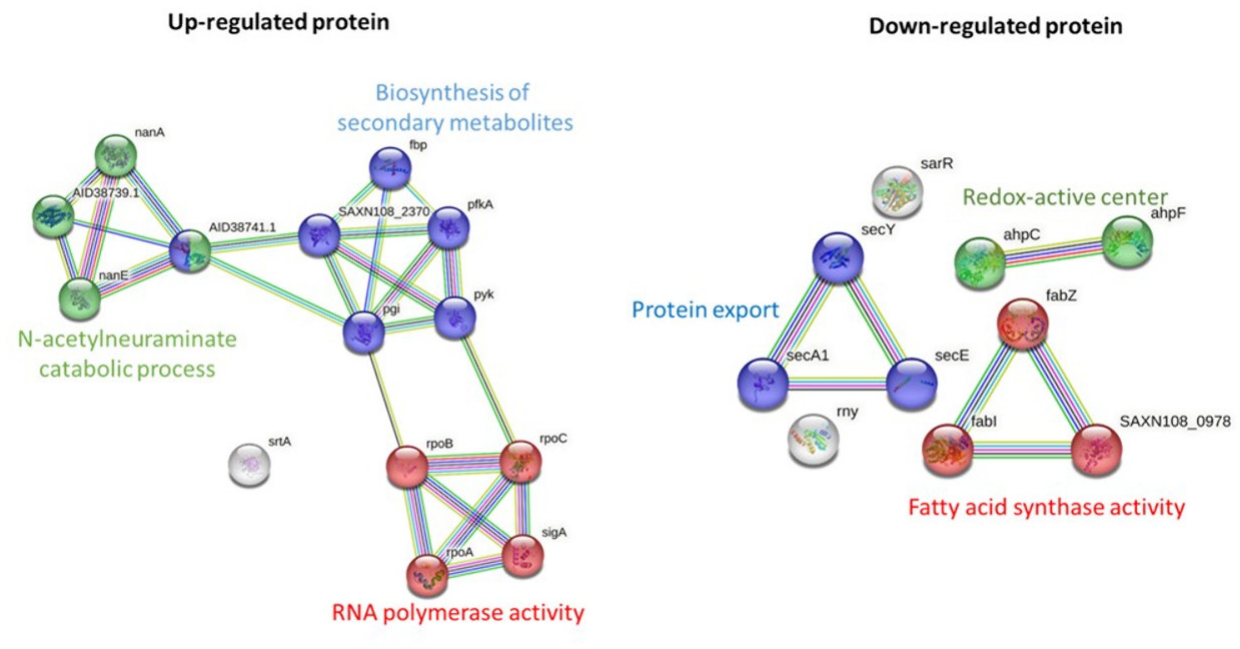
Pathway analysis of significantly upregulated and downregulated proteins in MRSA following *M. alba* treatment, conducted using the STRING database.

STRING-based pathway analysis indicated that upregulated proteins were associated with the N-acetylneuraminate catabolic process, RNA polymerase activity, and secondary metabolite biosynthesis. Downregulated pathways involved protein export, redox metabolism, and fatty acid biosynthesis (Fig. 3). All differential expression analyses were performed relative to solvent treated controls, ensuring that the observed changes are attributable to the *M. alba* extract rather than the solvents.

## Discussion

In this study, the ethanol extract of *M. alba* stems exhibited stronger antibacterial activity than leaf extracts, consistent with previous reports that stem tissues often contain higher concentrations of phenolic and flavonoid compounds (Wang et al., 2012; Batiha et al., 2023; D’Urso et al., 2019). Among the metabolites detected, betulinic acid was the most abundant, and together with flavonoids such as morin, luteolin, and quercetin, as well as stilbenoids like oxyresveratrol, morusin, and kuwanon G, these compounds are well known for their antibacterial and antioxidant properties (Wang et al., 2012; Faiz & Faiz, 2021; Škovranová et al., 2022; Zhu et al., 2024; Zuo et al., 2018). The higher potency of stem extracts compared to leaves likely reflects both the enrichment and the specific composition of these bioactive metabolites, which are influenced by tissue-specific biosynthesis.

Solvent selection also significantly affected antibacterial activity. Ethanol, a moderately polar organic solvent, efficiently extracts a broad range of polar to moderately nonpolar compounds, including flavonoid aglycones, phenolics, and triterpenoids such as betulinic acid, morusin, and kuwanon G (Batiha et al., 2023). In contrast, water preferentially extracts highly polar compounds, such as glycosides, polysaccharides, and certain phenolic acids, which may exhibit lower antibacterial potency. Consequently, ethanolic extracts showed stronger activity, while aqueous decoctions were generally weaker or inactive. The MIC, MBC, and time-kill assays confirmed that the ethanol stem extract exhibits measurable antibacterial effects against MRSA, although its potency is considerably lower than that of purified compounds or standard antibiotics. This reduced activity is expected for crude extracts, which contain complex mixtures of bioactive constituents at relatively low individual concentrations. Nonetheless, the presence of multiple active compounds may provide complementary or synergistic effects, contributing to the overall antibacterial response. The use of crude extract in this study was therefore intentional, aiming to preserve potential synergistic interactions among phytochemicals and to provide an initial screening of antibacterial potential prior to further purification and characterization. For future studies, it would be valuable to quantify the relative abundance of individual active compounds and systematically investigate their combinatorial effects. Such studies could clarify the contribution of each metabolite and provide deeper insights into the potential synergistic interactions that underlie the antibacterial activity of *M. alba* extracts.

Proteomic profiling of MRSA exposed to the *M. alba* stem extract revealed significant differential protein expression compared to solvent treated controls. Among upregulated proteins, the bifunctional protein GlmU, previously implicated in stress-induced biofilm formation (Di Somma et al., 2019), was markedly increased, potentially indicating a bacterial stress response aimed at survival (Sharma, Misba & Khan, 2019). The upregulation of N-acetylneuraminate lyase, an enzyme linked to bacterial colonization and a promising antibacterial drug target (North et al., 2016; Almagro-Moreno & Boyd, 2009), further supports this stress-adaptation hypothesis. Additionally, ribonuclease E, a protein involved in RNA processing and previously proposed as a target for antibacterial development (Mardle et al., 2020), was significantly upregulated, suggesting that RNA metabolism may be affected by the extract. In contrast, several key proteins were downregulated, including ribose-phosphate pyrophosphokinase, an enzyme essential to nucleotide biosynthesis (Visentin, Hasnain & Gallin, 1977; Lopatkin & Yang, 2021). This downregulation likely impairs nucleic acid synthesis, thereby inhibiting bacterial replication. SecA, a key ATPase involved in protein translocation and bacterial virulence (Chitlaru et al., 2006; Chaudhary et al., 2015), was also significantly reduced, indicating disruption of essential secretion systems. Furthermore, the downregulation of the ATP-dependent zinc metalloprotease FtsH, a protein quality control enzyme involved in degradation of damaged proteins (Gottesman, 1999; Fischer et al., 2002), may impair bacterial homeostasis and survival under stress conditions. By comparing protein expression to solvent-treated controls, we ensured that these changes reflect the specific effect of the plant extract rather than solvent-induced alterations.

Pathway enrichment analysis revealed that the N-acetylneuraminate catabolic process was among the most significantly upregulated pathways following treatment. This metabolic adaptation may reflect an attempt by MRSA to utilize alternative nutrient sources under stress (Jeong et al., 2009). The upregulation of pathways related to secondary metabolite biosynthesis and RNA polymerase activity suggests a broader bacterial response to mitigate the impact of the extract, likely by altering transcriptional and metabolic activities to promote survival. On the other hand, pathways associated with protein export, fatty acid biosynthesis, and redox-active center activity were significantly downregulated. The suppression of protein export, especially the Sec-dependent pathway, may reduce the secretion of virulence factors and detoxification proteins (Grkovic, Brown & Skurray, 2002; Jin et al., 2018). Downregulation of the redox-active center, a known mediator in bacterial competition and oxidative stress response (Dietrich et al., 2008), could render bacteria more vulnerable to oxidative damage. The observed suppression of fatty acid synthase activity, a critical target for several antibiotics such as triclosan and isoniazid (Wright & Reynolds, 2007), highlights a plausible mechanism through which the *M. alba* extract exerts its antibacterial effect.

Together, these findings provide a comprehensive view of the antibacterial potential of *M. alba* stem extract. The extract appears to interfere with critical bacterial pathways and proteins involved in metabolism, survival, and virulence. While the antibacterial activity of *M. alba* and its constituent betulinic acid has been reported previously, the present study provides new insights by combining comparative evaluation of plant parts and extraction solvents with mechanistic investigation using proteomic profiling. This approach enables the identification of MRSA protein targets and affected metabolic pathways, offering deeper understanding of the extract’s modes of action beyond the effects of individual compounds. Furthermore, the LC-QTOF-MS/MS profiling revealed a complex mixture of flavonoids, phenolics, and triterpenoids, suggesting possible synergistic interactions that collectively contribute to antibacterial efficacy. Our findings offer additional understanding of how *M. alba* extracts might influence MRSA, complementing prior research that examined individual compounds or simple antibacterial activities.

A limitation of this study is that betulinic acid was not quantified or tested in isolation, which should be addressed in future investigations. Although betulinic acid was identified as the most abundant metabolite, the relative contributions of other constituents such as morusin, kuwanon G, and quercetin remain to be determined. Future studies involving purified compounds or fractionated extracts will help clarify their individual and combined effects. Moreover, while the current work focused on antibacterial activity and proteomic responses, cytotoxicity assessment was not included. Evaluating cytotoxicity or hemolytic potential is essential to determine the selectivity and safety of the *M. alba* stem extract. Previous studies have shown that *M. alba* extracts generally exhibit low cytotoxicity toward mammalian cells at antibacterial concentrations (Yiemwattana, Chaisomboon & Jamdee, 2018; Batiha et al., 2023); however, further *in vitro* evaluation using standard cell viability or hemolysis assays is necessary to confirm the therapeutic window and ensure safety for potential topical or systemic applications.

## Conclusions

This study advances the understanding of how phytochemical-rich extracts from *M. alba* modulate bacterial physiology and identifies potential molecular targets in MRSA. The findings highlight the potential of *M. alba*-derived compounds as alternative or adjunctive therapeutic candidates for combating antibiotic-resistant bacterial infections.

## Supplemental Information

10.7717/peerj.20647/supp-1 Supplemental Information 1 A total of 138 metabolites were identified via LC-QTOF-MS/MS after filtering for quality and abundance

10.7717/peerj.20647/supp-2 Supplemental Information 2 Full-length, unprocessed SDS-PAGE gel image for Figure 2

10.7717/peerj.20647/supp-3 Supplemental Information 3 Raw CFU/mL counts used to generate the time-kill curve

## Additional Information and Declarations

### Field Study Permissions

The following information was supplied relating to field study approvals (i.e., approving body and any reference numbers): Leaves and stems of *Morus alba* were collected on 8 November 2022 from a cultivated tree at a private residence in Bang Kruai, Nonthaburi, Thailand (approximately 13.82°N, 100.39°E), with permission granted by Dr. Naowarat Saralamba, the landowner, cultivator of the plant, and corresponding author of this study. No specific field permit was required for sample collection from privately owned land.

### DNA Deposition

The following information was supplied regarding the deposition of DNA sequences: All the mass spectrometry raw data are available at the Science Data Bank repository: anonymous. 2024. “Unlocking the Antibacterial Power of *Morus alba* L. Against Methicillin-Resistant *Staphylococcus Aureus*.” Science Data Bank. doi: 10.57760/sciencedb.09514.

### Data Availability

The following information was supplied regarding data availability: The raw and processed data are available in the Supplementary Files.
