# Supplementary material for: Antibacterial and proteomic profiling of Morus alba extract against methicillin-resistant Staphylococcus aureus
Source: PeerJ. 2026 Jan 23;14:e20647. doi: 10.7717/peerj.20647 (PMC12834118; doi:10.7717/peerj.20647)
Supplement: Supplemental Information 1 [file peerj-14-20647-s001.pdf]

No.	#	Found At Mass	Area	Library Hit	Antimicrobial
1	1693	279.2439	119400000	10E,12Z-octadecadienoic acid	o
2	1605	455.3649	76860000	Betulinic acid	x
3	1503	409.2429	74450000	1-Hexadecanoyl-2-sn-glycero-3-phosphate	o
4	715	301.0447	64930000	Morin	x
5	608	243.0741	44120000	Oxyresveratrol	x
6	775	285.0467	30720000	Luteolin	x
7	1614	271.2318	28440000	2-Hydroxypalmitic acid	o
8	1602	277.2247	23550000	Pinolenic acid	o
9	275	339.0785	21950000	Esculin	x
10	1396	293.2138	19120000	9-Oxo-10(E),12(E)-octadecadienoic acid	x
11	1141	313.2409	14640000	12,13-Dihydroxy-9Z-octadecenoic acid	o
12	776	301.0375	14080000	Quercetin	x
13	378	177.0263	11250000	Esculetin	x
14	1223	323.1311	9949000	Bavachin	x
15	1432	483.2752	8226000	1-Palmitoyl-2-hydroxy-sn-glycero-3-phospho-(1'-rac-glycerol)	o
16	286	175.0653	7742000	2-Isopropylmalic acid	x
17	515	163.0427	7577000	p-Coumaric acid	x
18	1558	435.2584	7518000	1-Oleoyl-L-.alpha.-lysophosphatidic acid	o
19	100	191.0583	7195000	(-)-Quinic acid	x
20	1702	453.3403	6439000	Betulonicacid	o
21	660	187.1004	4546000	Azelaic acid	x
22	1324	571.2922	4405000	1-Hexadecanoyl-sn-glycero-3-phospho-(1'-myo-inositol) (NIST) [Smart Confirmation]	o
23	1284	271.2294	4399000	16-Hydroxyhexadecanoic acid	o
24	801	315.0536	4158000	3',4',5,7-Tetrahydroxy-3-methoxyflavone	o
25	501	463.0915	4110000	Spiraeoside	o
26	1387	471.3486	3625000	Hederagenin	x
27	1293	471.3478	3284000	Hederagenin	x
28	129	133.0154	3212000	L-Malic acid	x
29	840	271.0629	3171000	Naringenin	x
30	542	161.0262	3073000	7-Hydroxycoumarin	x
31	748	263.1299	3067000	Abscisic acid	x
32	223	153.0203	2789000	Protocatechuic acid	x
33	1431	421.1308	2547000	Mangiferin	x
34	1692	281.2406	2530000	(Z)-6-Octadecenoic acid	o

Note: Symbols for Antimicrobial Activity
o = No published paper reporting antimicrobial activity
x = Published paper reports antimicrobial activity

No.	#	Found At Mass	Area	Library Hit	Antimicrobial
35	587	449.1103	2278000	Deoxyrhapontin	o
36	861	285.0416	2259000	Kaempferol	x
37	331	353.09	2191000	Chlorogenic acid	x
38	848	269.0468	2034000	Apigenin	x
39	1385	293.1813	2011000	Myristyl sulfate	o
40	523	197.0463	1935000	Ethyl gallate	x
41	1111	161.0247	1826000	7-Hydroxycoumarin	x
42	312	567.1759	1818000	A Mulberroside A	x
43	1225	471.3483	1706000	Hederagenin	x
44	1732	313.2762	1673000	Phytol	x
45	165	117.02	1654000	Succinic acid	x
46	1200	265.1487	1603000	Dodecyl sulfate	o
47	131	188.0572	1602000	N-Acetyl-L-glutamic acid	o
48	1623	265.2183	1585000	Dodecyl sulfate	o
49	1346	455.3172	1409000	Oleanolic acid	x
50	285	137.0258	1375000	4-Hydroxybenzoic acid	x
51	704	227.0724	1327000	Resveratrol	x
52	520	205.0148	1248000	7-Hydroxycoumarin-3-carboxylic acid	o
53	556	463.0895	1241000	Spiraeoside	o
54	382	179.0356	1239000	Caffeic acid	x
55	799	207.0672	1182000	Ethyl trans-caffeate	o
56	1550	337.2057	1143000	Ethylene glycol tetradecyl ether sulfate	o
57	1256	723.3838	1128000	Gingerglycolipid B	o
58	1383	483.2732	1109000	1-Palmitoyl-2-hydroxy-sn-glycero-3-phospho-(1'-rac-glycerol)	o
59	961	322.2032	1106000	N-(-)-Jasmonoyl-(S)-isoleucine	o
60	761	185.0823	1066000	Endothal	o
61	1613	699.4996	1018000	1,2-Dioleoyl-sn-glycero-3-phosphate	o
62	654	269.0472	1002000	7,3',4'-Trihydroxyflavone	o
63	588	403.1047	996400	Deoxyrhapontin	o
64	1371	452.2783	969800	1-Palmitoyl-2-hydroxy-sn-glycero-3-phosphoethanolamine	o
65	568	193.0511	951300	Ferulic Acid	x
66	760	161.0246	945400	7-Hydroxycoumarin	x
67	1331	309.1749	934600	Ethylene glycol dodecyl ether sulfate	o
68	169	215.033	885300	D-Galactose	x
69	1494	509.2938	837000	1-Oleoyl-2-hydroxy-sn-glycero-3-phospho-(1'-rac-glycerol) (NIST) [Smart Confirmation]	o
70	670	317.0333	817600	Myricetin (NIST) [Smart Confirmation]	x
71	1126	487.3433	772700	Asiatic acid (NIST) [Smart Confirmation]	x
72	663	137.0249	769400	4-Hydroxybenzoic acid	x
73	1402	478.2938	746000	1-Oleoyl-sn-glycero-3-phosphoethanolamine	o

No.	#	Found At Mass	Area	Library Hit	Antimicrobial
74	1713	699.4987	746000	1,2-Dioleoyl-sn-glycero-3-phosphate	o
75	195	137.0248	735300	Salicylic acid	x
76	618	447.0941	730600	Astragalin	x
77	1386	353.2019	718000	Diethylene glycol dodecyl ether sulfate	o
78	1259	487.3432	708700	Asiatic acid	x
79	194	109.0298	705800	Resorcinol	x
80	383	197.0464	695300	Syringic acid	x
81	1181	297.1539	673100	Ricinoleic acid	x
82	1678	453.3407	666100	Betulonicacid	x
83	1589	453.3375	659900	Betulonicacid	o
84	1404	397.2265	623900	Allopregnanolone sulfate	o
85	1421	243.197	553600	2-Hydroxymyristic acid	x
86	67	181.0719	536900	D-Sorbitol	o
87	103	209.0672	526200	D-Saccharic acid	o
88	1280	579.2861	513900	arctiin	o
89	178	169.0146	494900	Gallic acid	x
90	381	167.0353	474100	Vanillic acid	x
91	259	181.0148	459200	4-Hydroxyisophthalic acid	x
92	1414	413.3056	448600	3-Oxo-4-cholestenoic acid	o
93	919	161.0246	409900	6-Hydroxycoumarin	x
94	1590	469.3328	389600	11-Keto-.beta.-boswellic acid	o
95	1808	269.25	383300	Heptadecanoic acid	x
96	1493	299.2026	382700	3-Hydroxyoctadecanoic acid	o
97	1144	367.0831	376900	Cyclocurcumin	x
98	1704	673.4835	364500	1-Palmitoyl-2-oleoyl-sn-glycero-3-phosphate	o
99	997	313.2389	355100	9,10-Dihydroxy-12Z-octadecenoic acid	o
100	724	269.046	352300	Sulfuretin	x
101	1017	299.0929	349700	3-Hydroxy-9,10-Dimethoxypterocarpan	o
102	565	191.0353	345500	Isoscopoletin	o
103	887	315.0517	344400	Isorhamnetin	x
104	313	613.1801	337300	Mulberroside A	o
105	175	130.0873	336400	Creatine	x
106	1622	747.5211	330700	1-Palmitoyl-2-oleoyl-phosphatidylglycerol	o
107	1390	597.306	326900	1-(9Z-Octadecenoyl)-sn-glycero-3-phospho-(1'-myo-inositol)	o
108	1005	323.1297	296100	Isobavachin	x
109	628	165.056	280900	Ethyl 4-hydroxybenzoate	o
110	218	115.0035	278500	Thiophene-2-thiol	o
111	263	137.0244	275400	4-Hydroxybenzoic acid	x
112	852	215.1294	268900	Undecanedioic acid	o
113	551	435.1301	262700	Polydatin	o

No.	#	Found At Mass	Area	Library Hit	Antimicrobial
114	865	299.0566	260900	Diosmetin	x
115	1850	112.9863	253300	DL-Ornithine	o
116	843	152.0354	252500	Norphenylephrine	o
117	1561	599.3209	248600	1-Octadecanoyl-sn-glycero-3-phospho-(1'-myo-inositol)	o
118	262	197.0462	246400	2,5-Dihydroxybenzaldehyde	x
119	491	355.1044	240200	4-Methoxycinnamic acid	x
120	290	138.0559	224200	3-Hydroxypicolinic acid	x
121	247	353.0887	206400	Neochlorogenic acid	x
122	343	191.0355	190800	Isoscopoletin	o
123	552	389.1248	189800	Polydatin	x
124	1951	177.0924	166000	3-tert-Butyl-2-hydroxybenzaldehyde	o
125	545	609.1472	163400	Rutin	x
126	411	121.0296	156700	3-Hydroxybenzaldehyde	x
127	856	227.1292	143500	trans-Traumatic acid	o
128	1880	699.5024	111900	1,2-Dioleoyl-sn-glycero-3-phosphate	o
129	1807	313.2753	93900	Phytol	x
130	1588	699.5006	87560	1,2-Dioleoyl-sn-glycero-3-phosphate	o
131	1822	373.2755	80100	Ginkgolic acid II	x
132	261	183.0299	75560	3-O-Methylgallic acid	o
133	1856	265.1494	72950	Dodecyl sulfate	o
134	1733	591.2631	55650	Pheophorbide a	x
135	1879	673.4864	47160	1-Palmitoyl-2-oleoyl-sn-glycero-3-phosphate	o
136	1673	623.253	20960	Neferine	x
137	402	352.858	16170	Chlorogenic acid	x
138	1405	471.3463	15150	Hederagenin	x
