# Supplementary material for: Antibacterial and proteomic profiling of Morus alba extract against methicillin-resistant Staphylococcus aureus
Source: PeerJ. 2026 Jan 23;14:e20647. doi: 10.7717/peerj.20647 (PMC12834118; doi:10.7717/peerj.20647)
Supplement: Supplemental Information 2 [file peerj-14-20647-s002.pdf]

Supplementary file 2. Full-length, unprocessed SDS-PAGE gel image for Figure 2

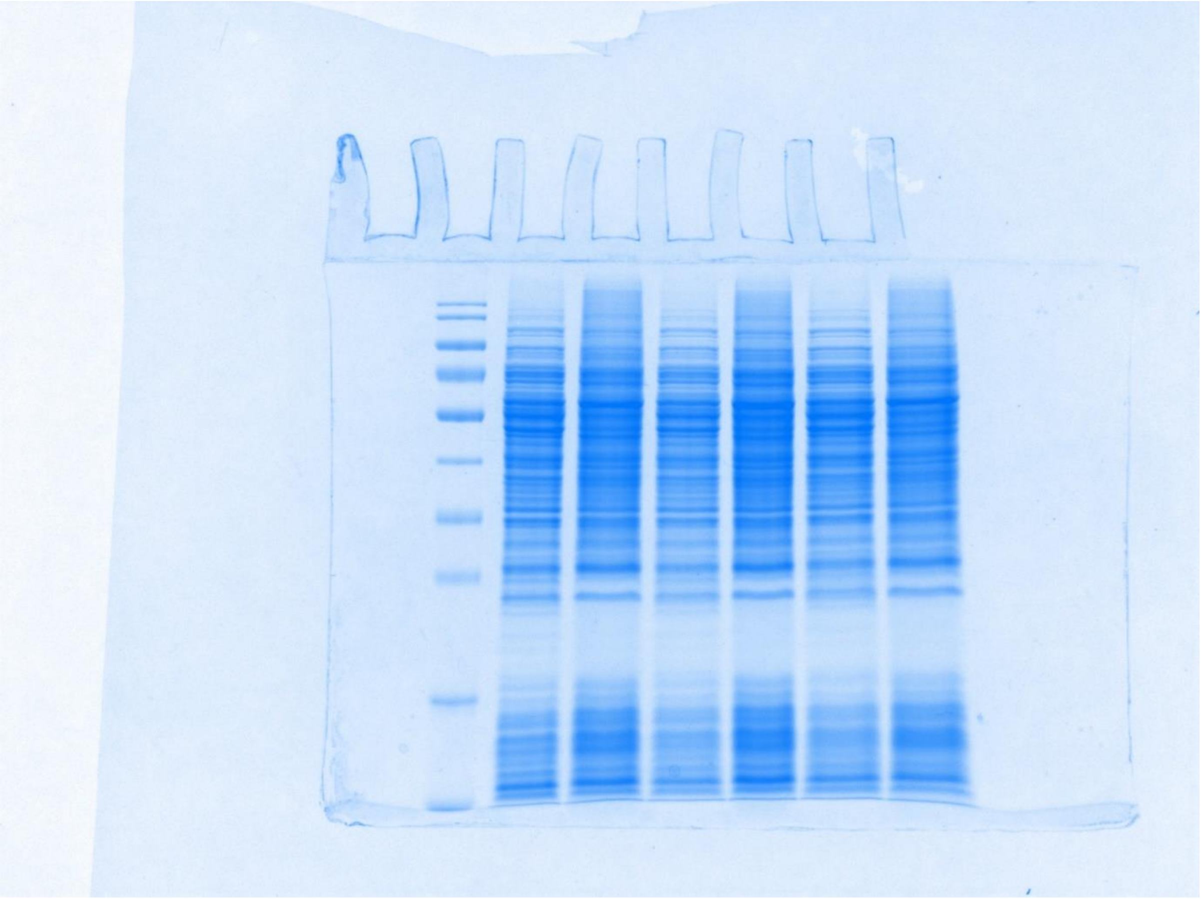

Full-length SDS-PAGE gel image showing protein profiles of MRSA treated with 0.1% DMSO (Control) and *M. alba* extract (Treatment) at IC50. This image corresponds to the cropped/greyscale-converted version shown in Figure 2 of the main manuscript. No contrast or exposure adjustments were applied to this original image.
